# Is There a Need to Reassess Protein Intake Recommendations Following Metabolic Bariatric Surgery?

**DOI:** 10.1007/s13679-025-00607-1

**Published:** 2025-01-29

**Authors:** Tair Ben-Porat, Yair Lahav, Tamara R. Cohen, Simon L. Bacon, Assaf Buch, Violeta Moizé, Shiri Sherf-Dagan

**Affiliations:** 1 School of Public Health, Faculty of Social Welfare and Health Sciences, University of Haifa, Haifa, Israel; 2 Department of Epidemiology and Preventive Medicine, School of Public Health, Sackler Faculty of Medicine and Sylvan Adams Sports Institute, Tel-Aviv, Israel; 3 Faculty of Land and Food Systems, Food, Nutrition and Health, University of British Columbia (UBC), Vancouver, BC Canada; 4 Montreal Behavioural Medicine Centre (MBMC), Centre Intégré Universitaire de Santé Et de Services Sociaux du Nord-de-L’Île-de-Montréal (CIUSSS-NIM), Montreal, QC Canada; 5 Department of Health, Kinesiology, and Applied Physiology, Concordia University, Montreal, QC Canada; 6 Department of Nutrition Sciences, School of Health Sciences, Ariel University, Ariel, Israel; 7 Institute of Endocrinology, Metabolism and Hypertension, Tel Aviv Sourasky Medical Center, Tel Aviv, Israel; 8 Obesity Unit, Hospital Clinic Barcelona, Barcelona, Spain; 9 Department of Nutrition, Tel-Aviv Assuta Medical Center, Tel Aviv, Israel

**Keywords:** Obesity, Metabolic Bariatric Surgery, Protein Requirements, Health outcomes

## Abstract

**Purpose of Review:**

Protein intake is recognized as a key nutritional factor crucial for optimizing Metabolic Bariatric Surgery (MBS) outcomes by preventing protein malnutrition, preserving fat-free mass, and inducing satiety. This paper discusses the current evidence regarding protein intake and its impact on clinical outcomes following MBS.

**Recent Findings:**

There are considerable gaps in the understanding of protein requirements following MBS, as existing guidelines are based on limited and inconsistent reports. This highlights the urgent need for updated clinical practice recommendations grounded in high-quality evidence.

**Summary:**

Further investigation using robust methodologies is essential to address existing research gaps related to the individualization of protein requirements following MBS. Future research should consider factors such as the time elapsed since surgery, the form and quantity of protein consumed, and necessary adjustments for physical activity. Ultimately, in alignment with recent literature, a more specific and personalized dietary protein approach should be examined.

## Introduction

Metabolic Bariatric Surgery (MBS) is the most effective long-term treatment for severe obesity and related medical conditions, but some patients may experience suboptimal postoperative health outcomes [1, 2]. Some factors that may lead to these suboptimal outcomes are low-quality dietary intake, maladaptive eating behaviors, and non-adherence to postoperative nutritional recommendations [3]. Specifically, protein intake (PI) could be of great importance to optimize postoperative outcomes, given protein effects on protein malnutrition prevention, fat-free mass (FFM) preservation, and the induction of satiety [1]. Several international societies have established clinical practice guidelines for minimal daily PI post-MBS [4–12], of which some based their recommendation on the AACE/TOS/ASMBS 2013 Guidelines [4, 5, 7, 10]. However, the scientific evidence supporting these recommendations is considered weak primarily due to a lack of objective and validated methods to assess protein requirements [2, 3, 13], and limited evaluation of its association with relevant clinical outcomes such as weight loss and body composition quality [2, 14, 15]. This perspective paper discusses the current evidence regarding relationships between PI and health-related outcomes following MBS; appropriate research designs and methodologies to evaluate protein requirements and MBS-relevant clinical outcomes; and the need for further data to make informed decisions on the recommended PI following MBS.

## Protein Intake and Absorption post-MBS

Following MBS, nutritional status is profoundly altered, particularly during the early postoperative period when food intake is dramatically decreased, leading to rapid changes in body weight, fat mass (FM), and lean body mass (LBM) [1, 2, 14, 15]. Several factors can lead to impaired PI and absorption post-MBS, including reduced gastric volume, changes in gut hormone levels, decreased secretion of hydrochloric acid and digestive enzymes, reduced surface area for nutrient absorption, and food intolerance or aversion to protein-rich foods [1, 2, 14, 15]. Protein malnutrition, characterized by hypoalbuminemia, anemia, edema, and alopecia, is a serious complication of malabsorptive procedures due to excessive malabsorption from bypassing large segments of the small intestine; however, protein depletion may also occur after restrictive procedures, primarily due to decreased intake during the early postoperative period, but also at a later stage due to surgical complications or maladaptive eating behaviors [15–17]. Given the importance of adequate PI post-MBS, several international associations have recommended a minimal intake of 60 g/day and up to 1.5 g/kg ideal body weight (IBW) per day (with up to 2.1 g/kg/day IBW in individual cases) [4, 5, 7, 10] Nevertheless, the methodology used across studies to create these recommendations often lacked validated and objective methods to evaluate the balance between PI and losses [13, 14, 18], as well as to examine the relationships between PI and specific clinical outcomes of MBS [2, 15, 19] (Table 1).

**Table 1:** Summary of available guidelines and sources of evidence on protein recommendations post-MBS

Guidelines	Recommendations for protein intake post-MBS	Grade of evidence	Source of the evidence utilized for crafting the recommendations within the guidelines
ASMBS Guidelines, 2008 [8]	A range of 60–80 g/day or 1.0–1.5 g/kg IBW per day, although the exact needs have yet to be defined Approximately 30% higher, equivalent to about 90 g/day after BPD-DS	Expert panel recommendations without formal evidence grading system	Clinical practice with a section detailing the etiology of potential deficiencies
Mechanick et al., 2013 and Mechanick et al., 2019 [4, 5]^1^	Minimum of 60 g/day and up to 1.5 g/kg IBW per day, with higher amounts of protein intake (up to 2.1 g/kg IBW per day) on an individualized basis In patients with severe protein malnutrition and/or hypoalbuminemia, not responsive to oral or EN protein supplementation, PN should be considered	Grade D (based on the AACE grading system)	Based on a narrative review, three original studies, and a conference abstract
Heber et al., 2010 [9]	An average of 60–120 g/day of protein to support lean body mass during weight loss, with especially importance in malabsorptive procedures, to prevent protein malnutrition and its effects	Strong recommendation with moderate quality evidence (based on GRADE grading system)	Clinical practice with a section detailing the etiology of potential deficiencies
Busetto et al., 2018 [7]	A minimal protein intake of 60 g/day and up to 1.5 g/kg IBW per day, with higher amounts of protein intake (up to 2.1 g/kg IBW per day) on an individualized basis.^1^ The use of liquid protein supplements (30 g/day) can facilitate adequate protein intake in the first period after surgery PN in case of severe non-responsive protein malnutrition	Grade D of recommendation with a low level of evidence (based on SIGN grading system)	Based on former guidelines, a narrative review, and two original studies
Stenberg et al., 2022 [6]	At least 60–80 g/day or 1.0–1.5 g/kg IBW per day following AGB, SG, and RYGB At least 90 g/day or as high as 2.1 g/kg IBW per day following malabsorptive procedures (SADI, OAGB, BPD-DS)	Not Specified	Based on a systematic review, a narrative review, and an original study
Vilallonga et al., 2019 [10]	Between 60–120 g/day of protein to maintain lean mass during weight loss.^1^	Grade B of Recommendation with a level of evidence 2a (based on Oxford classification)	Based on former guidelines, a narrative review, and four original studies
Shiau J et al., 2020 [12]	Range from 1.2 to 1.5 g/kg/day based on goal body weight A minimum of 60 g/day protein for SG/RYGB and 80–120 g/day for BPD-DS	Not Specified	Not Specified
Quilliot et al., 2021 [11]	A minimum of 60 g/day of protein and at least 1.1 g/kg IBW per day Protein supplementation should be added in case of failure to reach this target	Not Specified	Delphi method with a section detailing the rationale

**Abbreviations:** Adjustable Gastric Band (AGB), American Association of Clinical Endocrinologists (AACE), Biliopancreatic Diversion (BPD), Biliopancreatic Diversion with Duodenal Switch** (**BPD-DS), Enteral Nutrition (EN), Grading of Recommendations, Assessment, Development, and Evaluation (GRADE), Ideal Body Weight (IBW), Metabolic Bariatric Surgery (MBS), One Anastomosis Gastric Bypass (OAGB), Parenteral Nutrition (PN(, Roux-en-Y Gastric Bypass (RYGB), Single-Anastomosis Duodeno-Ileal Bypass (SADI), Scottish Intercollegiate Guidelines Network (SIGN), Sleeve Gastrectomy (SG)

^1^Mechanick et al. 2019, Busetto et al. 2018, and Vilallonga et al. 2019 based their recommendations on Mechanick et al., 2013

## Protein Intake and Clinical Outcomes Following MBS

### Weight Loss and Body Composition Changes

An extreme calorie deficit induced by surgery can compromise the ability to preserve muscle mass during rapid and extensive weight loss. The deterioration of skeletal muscle mass is driven by factors such as the extent of calorie restriction, age, inflammation, and anabolic resistance [20]. This eventually leads to a decline in muscle protein synthesis and increased muscle protein breakdown [14], implying that PI might need to be modulated during the post-MBS period [21]. High PI is hypothesized to be beneficial for weight, FM reduction, and FFM preservation through several mechanisms, including increased satiety and rate of energy expenditure, and the stimulation of anabolic muscle mass synthesis [3, 19]. The effects of higher PI, either through diet or supplementation, on optimizing weight loss and improving body composition quality, have been indicated by several randomized controlled trials (RCTs) in adults who practice resistance training as well as in patients who underwent nonsurgical caloric restrictions [2, 19]. Conversely, only a limited number of studies have assessed the effects of protein-enriched diets or protein supplementation on post-MBS weight outcomes and body composition changes, and these have reported inconsistent results [1, 2, 19]. A recent systematic review of eight studies (i.e., prospective and retrospective cohorts, cross-sectional, and nonrandomized clinical trials, n = 2,378) found greater PI to be associated with better weight outcomes after MBS [3]. In contrast, a 2021 systematic review of five RCTs (n = 223), showed no advantage of protein supplementation or a protein-enriched diet (≥ 60 g/day) on weight outcomes post-MBS and provided inconclusive evidence regarding their benefit for LBM preservation [22]. Nevertheless, the latest published meta-analysis which synthesized eight RCTs (n = 341), indicated that PI higher than the generally recommended value of 0.8 g/kg/day can lead to greater weight and FM loss post-MBS compared to a normal protein diet, with FFM preservation seen only among patients undergoing sleeve gastrectomy (SG) consuming at least 40 g/ day in addition to the daily recommended value [19]. Of note, it is possible to assume that PI in most experiments was insufficient to preserve FFM, as two of the included studies that applied higher amounts of protein (i.e., 1.2 g/kg IBW/ day and 2 g/kg IBW/ day) demonstrated significantly increased postoperative FFM, whereas in others, FFM decreased or remained unchanged [19].

### Nutritional Status and Protein Deficiency Post-MBS

Protein deficiency remains the most severe macronutrient complication associated with MBS, manifesting as protein malnutrition or protein-energy malnutrition, and can be developed even several years post-MBS [15]. Protein deficiency can be caused by prolonged low-quality dietary intake and malabsorption, being frequently characterized by low albumin and prealbumin levels, unexplained anemia, and edema [15, 17]. Most studies that assessed blood albumin and/or prealbumin levels post-MBS found albumin levels to be in the normal range after SG or Roux-en-Y Gastric Bypass (RYGB), and significantly lower prealbumin levels during the early postoperative period of these procedures, but within safe limits [2]. Although albumin and prealbumin are commonly used to assess protein status in MBS, they are not sensitive markers of PI adequacy and should not guide protein requirement recommendations for patients following MBS [2, 15, 23].

### Protein Intake and Skeletal Health Post-MBS

Bone mineral density, especially in the femoral neck, decreases after MBS [1, 15]; Only a single study to date has reported a significant positive association between protein supplementation and bone markers, however, this study incorporated multiple interventions simultaneously besides protein supplements [24].

## Methodological Considerations of Protein Requirements Evaluation in MBS

Current international recommendations are not based on studies specifically designed to assess protein requirements, e.g., utilizing techniques such as nitrogen balance, amino acid oxidation, or stable isotope tracers from substrate-specific to double-labeled water [13], but rather relying mostly on clinical outcomes like postsurgical serum protein markers and body composition alterations [25–27]. Importantly, a few recent studies have utilized nitrogen balance to assess protein requirements in patients following MBS [14, 18]. Guillet et al. evaluated nitrogen balance during the first year post-SG and RYGB, revealing a negative balance between nitrogen intake and losses in urine and stool, suggesting that spontaneous PI is insufficient to meet protein requirements for the majority of patients [14]. Similarly, Vinjamuri et al. evaluated nitrogen balance six months post-SG and RYGB, revealing a negative balance between nitrogen intake and urine nitrogen excretion, also indicating that dietary PI is inadequate in these individuals [18]. When discussing key methods to establish protein requirements in the MBS population, it is important to highlight the methodological limitations of common self-reported dietary intake assessment tools, which are subject to reporting bias and may influence the evidence levels of studies in this area [1, 2, 18]. Finally, in addition to the issue of conflicting measures used as indicators for sufficient PI in MBS, additional methodological limitations of the available evidence regarding protein requirements following MBS evaluation need to be acknowledged. When assessing the impact of sufficient PI on body composition, it is crucial to consider that regional and total body composition can be estimated using various techniques (e.g., Magnetic resonance imaging, Dual-Energy X-ray Absorptiometry, Bioelectrical Impedance Analysis, and air displacement plethysmography), which could influence results [2, 19, 22]. Additionally, functional testing (e.g., handgrip and sit-stand tests) to evaluate the relationship between FFM changes and muscle strength might also be beneficial for future studies [18]. It is also critical to evaluate adherence to interventions aimed at increasing PI, as adherence tends to decline over time, often failing to reach the recommended intake levels set at the beginning of the study, thereby potentially impacting outcomes [2, 19, 22]. To enhance participant engagement and adherence to PI interventions, researchers should consider designing their studies using validated behavioural intervention frameworks [28]. In parallel, the form of protein and the effectiveness of protein supplementation need to be further examined [2, 19, 22].

Most studies evaluating the impact of PI on clinical outcomes in MBS did not assess the level and type of physical activity, despite the known significant role that it plays in the preservation of muscle mass and strength [19, 22]. Studies investigating the role of PI on clinical outcomes should distinguish between the short-term post-surgery periods, characterized by extensive weight loss, and the longer-term post-surgery periods, which include phases of weight stability or regain [3]. Lastly, studies should also differentiate between types of MBS and focus on specific procedures to tailor specific PI recommendations.

## Need for Further Investigation: A Call to Scientific Community

**Fig. 1:**
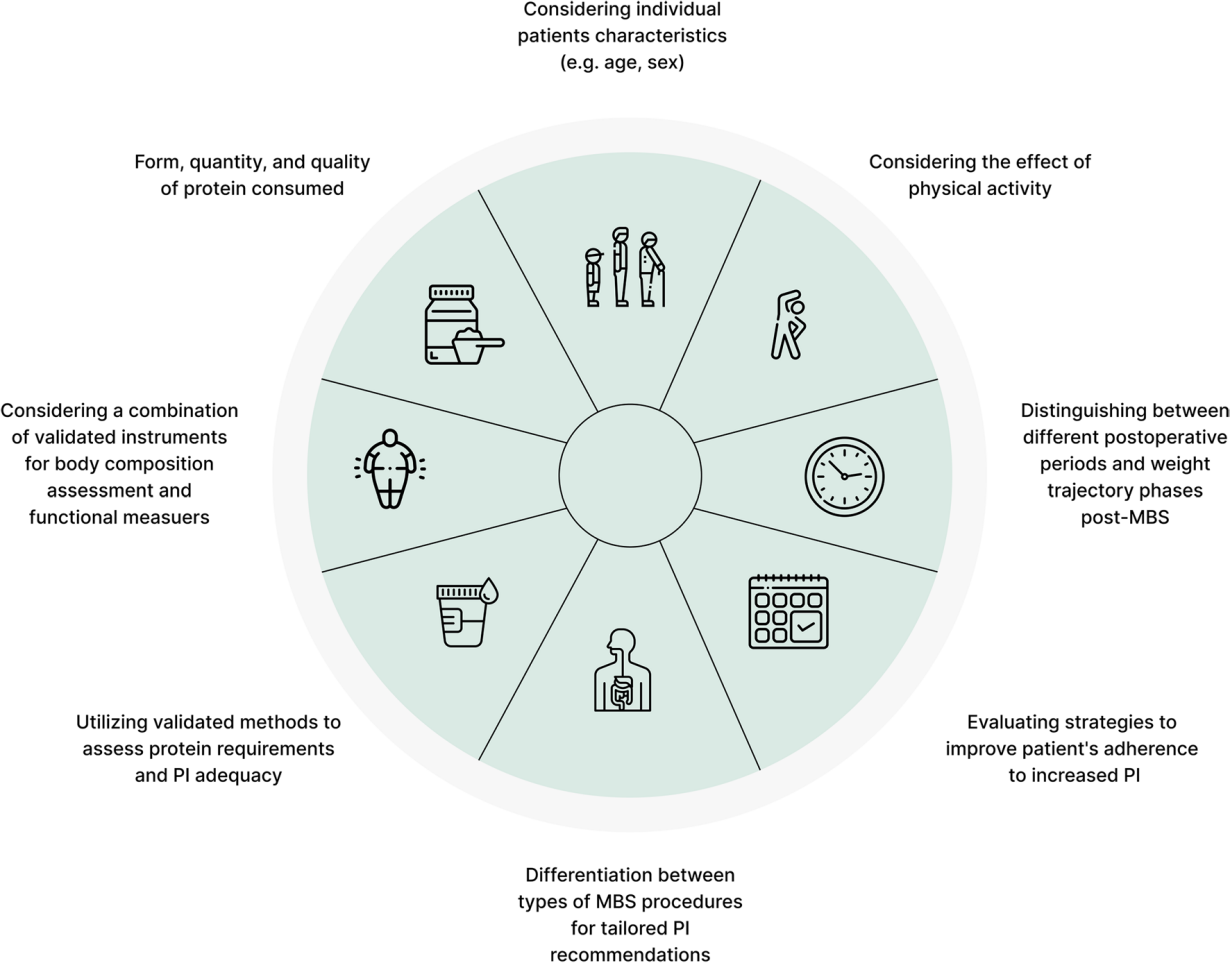
Key considerations for conducting research to evaluate dietary protein needs following MBS. Abbreviation: Metabolic Bariatric Surgery (MBS); Protein Intake (PI)

A thorough investigation into defining dietary protein needs following MBS is essential, as these requirements have yet to be established using robust and validated methods [2, 13, 14, 18, 22]. Recent studies employing better methods suggest that dietary PI in the short-term post-MBS may not suffice to meet patients’ requirements and underscores the need for a more personalized dietary protein recommendations approach [14, 18, 22]. Findings also highlight the importance of differentiating protein needs at various stages post-MBS, particularly during rapid weight loss, but also at later stages of weight stabilization and weight regain prevention [2, 14, 18, 22]. Collectively, high-quality research is necessary to establish individual recommendations and to update clinical practice guidelines for PI, addressing the aforementioned methodological limitations and incorporating factors such as the form, quantity, and quality of protein consumed, the combined effect of physical activity, across larger populations and longer follow-up durations [2, 14, 18, 19, 22]. Figure 1 illustrates key considerations in the research implementation on dietary protein needs following MBS.

## Data Availability

No datasets were generated or analysed during the current study.

## Abbreviations

BMI: Body mass index
FFM: Fat free mass
LBM: Lean body mass
MBS: Metabolic bariatric surgery
RCT: Randomized controlled trial

## References

[1] van den Broek M, de Heide LJ, Veeger NJ, van der Wal-Oost AM, van Beek AP. Influence of dietary protein and its amino acid composition on postoperative outcomes after gastric bypass surgery: a systematic review. Nutr Rev. 2016;74:749–73. 27864536 10.1093/nutrit/nuw042

[2] Ito MK, Gonçalves VSS, Faria SLCM, et al. Effect of protein intake on the protein status and lean mass of post-bariatric surgery patients: a systematic review. Obes Surg. 2017;27:502–12. 27844254 10.1007/s11695-016-2453-0

[3] de Souza Vilela DL, da Silva A, Pinto SL, Bressan J. Relationship between dietary macronutrient composition with weight loss after bariatric surgery: A systematic review. Obes Rev. 2023;24:e13559. 36890787 10.1111/obr.13559

[4] Mechanick JI, Youdim A, Jones DB, et al. Clinical practice guidelines for the perioperative nutritional, metabolic, and nonsurgical support of the bariatric surgery patient—2013 update: cosponsored by American Association of Clinical Endocrinologists, the Obesity Society, and American Society for Metabolic & Bariatric Surgery. Surg Obes Relat Dis. 2013;9:159–91. 23537696 10.1016/j.soard.2012.12.010

[5] Mechanick JI, Apovian C, Brethauer S, et al. Clinical practice guidelines for the perioperative nutrition, metabolic, and nonsurgical support of patients undergoing bariatric procedures–2019 update: cosponsored by American Association of Clinical Endocrinologists/American College of Endocrinology, The Obesity Society, American Society for Metabolic & Bariatric Surgery, Obesity Medicine Association, and American Society of Anesthesiologists. Surg Obes Relat Dis. 2020;16:175–247. 31917200 10.1016/j.soard.2019.10.025

[6] Stenberg E, dos Reis Falcao LF, O’Kane M, et al. Guidelines for perioperative care in bariatric surgery: Enhanced Recovery After Surgery (ERAS) society recommendations: a 2021 update. World J Surg. 2022;46:729–51. 10.1007/s00268-021-06394-9 PMC8885505 34984504

[7] Busetto L, Dicker D, Azran C, et al. Practical recommendations of the obesity management task force of the European association for the study of obesity for the post-bariatric surgery medical management. Obes Facts. 2018;10:597–632. 10.1159/000481825 PMC5836195 29207379

[8] Aills L, Blankenship J, Buffington C, Furtado M, Parrott J. ASMBS allied health nutritional guidelines for the surgical weight loss patient. Surg Obes Relat Dis. 2008;4:S73–108. 18490202 10.1016/j.soard.2008.03.002

[9] Heber D, Greenway FL, Kaplan LM, et al. Endocrine and nutritional management of the post-bariatric surgery patient: an Endocrine Society Clinical Practice Guideline. J Clin Endocrinol Metab. 2010;95:4823–43. 21051578 10.1210/jc.2009-2128

[10] Vilallonga R, Pereira-Cunill J, Morales-Conde S, et al. A Spanish Society joint SECO and SEEDO approach to the Post-operative management of the patients undergoing surgery for obesity. Obes Surg. 2019;29:3842–53. 31342249 10.1007/s11695-019-04043-8

[11] Quilliot D, Coupaye M, Ciangura C, et al. Recommendations for nutritional care after bariatric surgery: Recommendations for best practice and SOFFCO-MM/AFERO/SFNCM/expert consensus. J Visc Surg. 2021;158:51–61. 33436155 10.1016/j.jviscsurg.2020.10.013

[12] Shiau J, Biertho L. Canadian Adult obesity clinical practice guidelines: bariatric surgery: postoperative management. Downloaded from: https://obesitycanada.ca/guidelines/postop. Accessed 24 Jan 2025

[13] Joint WHO/FAO/UNU Expert Consultation. Protein and amino acid requirements in human nutrition. World Health Organization technical report series. 2007;935:1–265. 18330140

[14] Guillet C, Masgrau A, Mishellany-Dutour A, et al. Bariatric surgery affects obesity-related protein requirements. Clin Nutr ESPEN. 2020;40:392–400. 33183568 10.1016/j.clnesp.2020.06.007

[15] Ben-Porat T, Elazary R, Sherf-Dagan S, et al. Bone health following bariatric surgery: implications for management strategies to attenuate bone loss. Adv Nutr. 2018;9:114–27. 29659692 10.1093/advances/nmx024 PMC5916426

[16] Lupoli R, Lembo E, Saldalamacchia G, et al. Bariatric surgery and long-term nutritional issues. World J Diabetes. 2017;8:464. 29204255 10.4239/wjd.v8.i11.464 PMC5700383

[17] O’Kane M, Parretti HM, Pinkney J, et al. British Obesity and Metabolic Surgery Society Guidelines on perioperative and postoperative biochemical monitoring and micronutrient replacement for patients undergoing bariatric surgery—2020 update. Obes Rev. 2020;21:e13087. 32743907 10.1111/obr.13087 PMC7583474

[18] Vinjamuri RG, Wu V, Eng A, et al. The impact of bariatric surgery on nitrogen balance at six months post-surgery. Obes Surg. 2024;34:2363–8. 38748346 10.1007/s11695-024-07269-3

[19] Golzarand M, Toolabi K, Mirmiran P. The effects of protein intake higher than the recommended value on body composition changes after bariatric surgery: A meta-analysis of randomized controlled trials. Clin Nutr. 2024;43:708–18. 38320462 10.1016/j.clnu.2024.01.031

[20] Carbone JW, McClung JP, Pasiakos SM. Recent advances in the characterization of skeletal muscle and whole-body protein responses to dietary protein and exercise during negative energy balance. Adv Nutr. 2019;10:70–9. 30596808 10.1093/advances/nmy087 PMC6370268

[21] Smeuninx B, Mckendry J, Wilson D, Martin U, Breen L. Age-related anabolic resistance of myofibrillar protein synthesis is exacerbated in obese inactive individuals. J Clin Endocrinol Metab. 2017;102:3535–45. 28911148 10.1210/jc.2017-00869 PMC5587073

[22] Romeijn MM, Holthuijsen DD, Kolen AM, et al. The effect of additional protein on lean body mass preservation in post-bariatric surgery patients: a systematic review. Nutr J. 2021;20:1–9. 33715633 10.1186/s12937-021-00688-3 PMC7958440

[23] Evans DC, Corkins MR, Malone A, et al. The use of visceral proteins as nutrition markers: an ASPEN position paper. Nutr Clin Pract. 2021;36:22–8. 33125793 10.1002/ncp.10588

[24] Muschitz C, Kocijan R, Haschka J, et al. The impact of vitamin D, calcium, protein supplementation, and physical exercise on bone metabolism after bariatric surgery: the BABS study. J Bone Miner Res. 2016;31:672–82. 26350034 10.1002/jbmr.2707

[25] Moizé V, Andreu A, Rodríguez L, et al. Protein intake and lean tissue mass retention following bariatric surgery. Clin Nutr. 2013;32:550–5. 23200926 10.1016/j.clnu.2012.11.007

[26] Raftopoulos I, Bernstein B, O’Hara K, et al. Protein intake compliance of morbidly obese patients undergoing bariatric surgery and its effect on weight loss and biochemical parameters. Surgery for Obesity and Related Diseases. 2011;7:733–42. 21925961 10.1016/j.soard.2011.07.008

[27] Andreu A, Moizé V, Rodríguez L, Flores L, Vidal J. Protein intake, body composition, and protein status following bariatric surgery. Obes Surg. 2010;20:1509–15. 20820937 10.1007/s11695-010-0268-y

[28] Bacon SL, Lavoie KL, Ninot G, et al. An international perspective on improving the quality and potential of behavioral clinical trials. Curr Cardiovasc Risk Rep. 2015;9:1–6.

